# Prion propagation estimated from brain diffusion MRI is subtype dependent in sporadic Creutzfeldt–Jakob disease

**DOI:** 10.1007/s00401-020-02168-0

**Published:** 2020-06-13

**Authors:** Riccardo Pascuzzo, Neil P. Oxtoby, Alexandra L. Young, Janis Blevins, Gianmarco Castelli, Sara Garbarino, Mark L. Cohen, Lawrence B. Schonberger, Pierluigi Gambetti, Brian S. Appleby, Daniel C. Alexander, Alberto Bizzi

**Affiliations:** 1 Neuroradiology Unit, Fondazione IRCCS Istituto Neurologico Carlo Besta, Via Celoria, 11, 20133 Milan, Italy; 2 Centre for Medical Image Computing, Department of Computer Science, University College London, London, UK; 3 Department of Neuroimaging, Institute of Psychiatry, Psychology and Neuroscience, King′S College London, London, UK; 4 National Prion Disease Pathology Surveillance Center, Case Western Reserve University, School of Medicine, Cleveland, OH USA; 5 Methods for Image and Data Analysis Group, Dipartimento di Matematica, Università Degli Studi di Genova, Genoa, Italy; 6 Department of Pathology, Case Western Reserve University, School of Medicine, Cleveland, OH USA; 7 Department of Neurology, Case Western Reserve University, University Hospitals Cleveland Medical Center, Cleveland, OH USA; 8 National Center for Emerging and Zoonotic Infectious Diseases, Centers for Disease Control and Prevention, Atlanta, GA USA; 9 Department of Psychiatry, Case Western Reserve University, University Hospitals Cleveland Medical Center, Cleveland, OH USA

**Keywords:** Disease progression, Prion propagation, Epicentre, Spongiform degeneration, Prion disease, Creutzfeldt–Jakob disease, Magnetic resonance imaging

## Abstract

Sporadic Creutzfeldt–Jakob disease (sCJD) is a transmissible brain proteinopathy. Five main clinicopathological subtypes (sCJD-MM(V)1, -MM(V)2C, -MV2K, -VV1, and -VV2) are currently distinguished. Histopathological evidence suggests that the localisation of prion aggregates and spongiform lesions varies among subtypes. Establishing whether there is an initial site with detectable imaging abnormalities (epicentre) and an order of lesion propagation would be informative for disease early diagnosis, patient staging, management and recruitment in clinical trials. Diffusion magnetic resonance imaging (MRI) is the most-used and most-sensitive test to detect spongiform degeneration. This study was designed to identify, in vivo and for the first time, subtype-dependent epicentre and lesion propagation in the brain using diffusion-weighted images (DWI), in the largest known cross-sectional dataset of autopsy-proven subjects with sCJD. We estimate lesion propagation by cross-sectional DWI using event-based modelling, a well-established data-driven technique. DWI abnormalities of 594 autopsy-diagnosed subjects (448 patients with sCJD) were scored in 12 brain regions by 1 neuroradiologist blind to the diagnosis. We used the event-based model to reconstruct sequential orderings of lesion propagation in each of five pure subtypes. Follow-up data from 151 patients validated the estimated sequences. Results showed that epicentre and ordering of lesion propagation are subtype specific. The two most common subtypes (-MM1 and -VV2) showed opposite ordering of DWI abnormality appearance: from the neocortex to subcortical regions, and vice versa, respectively. The precuneus was the most likely epicentre also in -MM2 and -VV1 although at variance with -MM1, abnormal signal was also detected early in cingulate and insular cortices. The caudal-rostral sequence of lesion propagation that characterises -VV2 was replicated in -MV2K. Combined, these data-driven models provide unprecedented dynamic insights into subtype-specific epicentre at onset and propagation of the pathologic process, which may also enhance early diagnosis and enable disease staging in sCJD.

## Introduction

Prion diseases are devastating neurodegenerative disorders. They may occur sporadically, be inherited, or acquired from dietary or iatrogenic exposure to prions. Despite their rarity, prionopathies are the focus of continued interest from the scientific community, because researchers have shown that a prion-like formation mechanism might also apply to other cellular proteins associated with major neurodegenerative diseases such as Alzheimer and Parkinson diseases [25].

Sporadic Creutzfeldt–Jakob disease (sCJD), the most common type of prion disease, accounts for 85–90% of all human cases [34]. Familial or genetically determined CJD (gCJD), the second most common type, accounts for an additional ~ 10% of the total, while the remainder includes very rare sporadic and familial types of fatal insomnia (sFI and FFI), variably protease-sensitive prionopathy (VPSPr) and Gerstmann–Sträussler–Scheinker (GSS) as well as the form acquired by infection. Sporadic CJD comprises several clinically and histopathologically distinct subtypes. This heterogeneity is largely encoded by the pairings of the patient genotype at the methionine (M) and valine (V) polymorphic codon 129 (which determines the MM, MV, and VV genotypes, henceforth denoted PRNP129 genotype) with the type (1 or 2) of the misfolded, disease-related prion protein (PrP^D^) [13, 26]. The prevalence of these associations suggests that the PRNP129 genotype favours the selection of a distinct PrP^D^ type that in turn determines the phenotype [13]. Currently, five pure clinicopathological subtypes of sCJD are recognized: sCJD-MM(V)1, -MM(V)2C, -MV2K, -VV1, and -VV2. The suffix C refers to spongiform degeneration with large vacuoles affecting preferentially the cortex. The suffix K refers to the presence of kuru plaques in the cerebellum. Approximately, 35% of sCJD cases show the co-occurrence of PrP^D^ types 1 and 2 [5]. Despite this complexity, the molecular classification of sCJD subtypes based on genotype-PrP^D^ type is accepted and used worldwide [4, 6, 19, 20, 22, 34].

All sporadic prion diseases are believed to be triggered by the spontaneous, and likely age-related, formation of misfolded PrP^D^, which replicates by seeded conversion using normal prion protein as a substrate [24]. The newly converted PrP^D^ would then spread and accumulate preferentially in selected brain regions where brain deposition of neurotoxic PrP^D^ typically causes three main histopathological lesions: vacuolation; astroglial and microglial activation; synaptic and neuronal loss. The postmortem histopathological observation that brain topography of spongiform degeneration and patterns of PrP^D^ deposition largely align with sCJD subtype suggests that site of initial formation and subsequent propagation of PrP^D^ are different in individual sCJD subtypes. However, to our knowledge, currently, no factual evidence is available on these two important aspects of the pathogenesis. Understanding in detail early pathogenic events of human prion diseases is essential not only to unravel etiologic mechanism but also because successful therapeutics will likely depend on early and accurate diagnosis and might need to be tailored to the prion disease subtype. However, accurate and detailed study of disease onset and propagation requires faithful modelling of sCJD subtypes that is currently unavailable.

Due to the high and early propensity to detect brain lesions, along with excellent diagnostic performance, diffusion magnetic resonance imaging (MRI) is an ideal tool for investigating initial PrP^D^-related lesions in vivo and their subsequent propagation in sCJD subtypes [1, 27, 32, 33]. However, the aggressive nature of most prion diseases severely limits the collection of longitudinal data. This thwarts traditional regression-based approaches for inferring propagation patterns.

Longitudinal patterns of disease progression can also be estimated from cross-sectional data by event-based modelling [11]. This method was first applied on patients with familial Alzheimer and Huntington diseases to identify orderings of regional brain atrophy, and, subsequently, to describe progression of other neurological diseases with the use of different types of biomarkers [8, 10, 18, 28, 31]. The instrinsic flexibility and the requirement of only cross-sectional data make the event-based model especially suitable for identifying the regional origin and propagation patterns in sCJD subtypes.

In this study, we used diffusion weighted imaging (DWI) and event-based modelling to determine in vivo the putative seed region where the disease starts, which we refer to here as the ‘epicentre’ and the ordering of lesion propagation in sCJD subtypes, while taking advantage of a large cohort of subjects with autopsy-confirmed sCJD and controls [1]. The event-based model provides a novel and precise numerical staging system in sCJD [31].

## Materials and methods

### Patients

The MRIs of patients with suspected prion disease referred to the National Prion Disease Pathology Surveillance Center (NPDPSC, Cleveland, Ohio, USA) were collected as part of a consultation service [1]. Subjects were included in this study if they had one positive DWI study and autopsy performed at NPDPSC. Subjects were assigned to sCJD group if they had confirmed diagnosis of one of seven pure sCJD molecular subtypes at brain autopsy, or to the control group if they had autopsy-confirmed diagnosis of non-prion disease and negative MRI. Twenty-five patients with pure sCJD subtype and negative MRI exam were excluded, because negative MRI are not informative for disease progression.

### Neuropathology

All diagnostic studies of brain tissue were performed at the NPDPSC and included: extensive histopathological and PrP^D^ immunohistochemical examinations as well as PrP gene and proteinase K-resistant PrP^D^ western blot analyses designed to achieve the final diagnosis of sCJD subtype. Subtype diagnosis of CJD was established as per criteria by Parchi and colleagues [20, 34]. “Pure subtype” of sCJD was defined by the detection of proteinase K-resistant PrP^D^ belonging to only one type following the examination of three brain regions and the exclusive presence of the corresponding neuropathological phenotype. Mixed subtypes showed the coexistence of both PrP^D^ types 1 and 2 (1–2) varying between 10 and 90% in the same brain region or occurring separately in different brain regions as well as presence of the corresponding neuropathological phenotypes.

### Diffusion MRI analysis

We considered 12 brain regions that are known to be selectively vulnerable to misfolded prion proteins [15]: 5 cerebral cortical regions (cortical ribbon of the frontal, temporal, occipital and parietal lobes, and the precuneus that was scored separately from the dorsal aspect of the parietal cortex), 3 regions of the limbic system (cingulate gyrus, insula, and head of hippocampus that includes the entorhinal cortex), the striatum (caudate and putamen), thalamus and cerebellum. The DWI signal hyperintensity in these brain regions were considered as separate candidate biomarkers to describe disease progression in all subjects.

An expert neuroradiologist (AB, with more than 15 years of experience) blind to clinical data, CSF laboratory results, and diagnosis, scored all DWI examinations using a semi-quantitative procedure established in a prior study [1]. Inter-rater reliability of this procedure with 2 other senior and one junior neuroradiologists was previously assessed in 200 patients [1]: it was excellent for 6 of 12 regions (intra-class correlation coefficient between 0.86 and 0.93), it was good for temporal cortex (0.83), cingulate and insula (0.81), occipital cortex (0.77), and cerebellum (0.70), and it was fair for the hippocampus (0.58). The neuroradiologists scored the DWI signal hyperintensities of the 12 brain regions on a 4-point ordinal scale: “zero” for no hyperintensity; “one” for questionable; “two” for CJD-related hyperintensity associated with low diffusivity on apparent diffusion coefficient maps; “three” for presence of extensive CJD-related hyperintensity with low diffusivity.

### Statistical analysis

In this section, we briefly describe the event-based model [11] and the modelling choices adopted here. The detailed formulation and estimation procedure are reviewed in the supplementary materials. The event-based model is a data-driven approach to estimating a longitudinal model of progression using only cross-sectional data that has been applied across multiple neurodegenerative diseases [8, 10, 11, 18, 28, 31]. Disease progression is described as a probabilistic sequence of events reflecting cumulative abnormality of disease biomarkers. The event-based model finds the most likely ordering of events by maximizing the data likelihood computed from the biomarker measurements of the subjects. We considered a set of 12 events corresponding to the transition from negative to positive visual grading of the MRI in the regions of interest defined above.

Implicitly, no events have occurred in controls, whereas we do not know, a priori, whether any given event has occurred in patients, because they are at various unknown degrees of disease severity (i.e., extent of lesion accumulation). Thus, patient biomarkers can be labelled as either normal (pre-event) or abnormal (event). These combinations of pre- and post-event inform the sequence through a discrete-distribution mixture model fit to patients and controls, with parameters of the pre-event component fixed after fitting to only data from the controls [11]. We chose a Bernoulli distribution to model the pre-event distribution, because all controls had a negative MRI (all regions scored 0 or 1). For the post-event distribution, we chose a uniform discrete distribution with support on the set {0, 1, 2, 3}. We fitted the mixture models using baseline data and used the limited longitudinal data we had to assess model consistency (see “Longitudinal validation” below) [11, 31].

Motivated by understanding lesion spread in the spectrum of sCJD, we built an event-based model for each of the seven pure sCJD subtypes, -MM1, -MM2 (also referred to as -MM2C), -MV1, -MV2C, -MV2K, -VV1, and -VV2. Moreover, we visualised the variability of the most likely ordering through the positional variance diagram obtained from Markov Chain Monte Carlo sampling [11], where the entry $\left(j,k\right)$ was the fraction of samples in which the *j*-th biomarker appeared in the *k*-th position of the sequence. To facilitate ease of comparison across subtypes, all the diagrams listed the brain macroregions in the following order: neocortex, limbic structures, striatum, thalamus, and cerebellum.

As is standard in event-based modelling, we provided a conservative estimate of uncertainty to test the robustness of the ordering by calculating new maximum likelihood event sequences for 100 bootstrapped data samples [8, 10, 11, 18, 28, 31].

We compared and contrasted the event-based models of subtypes qualitatively and quantitatively. Statistical overlap of two positional variance diagrams ${D}_{1/2}$ was quantified using the Bhattacharyya coefficient, averaged over events:

$$B\left( {D_{1} ,D_{2} } \right) = \frac{1}{12}\mathop \sum \limits_{j = 1}^{12} \mathop \sum \limits_{k = 1}^{12} \sqrt {D_{1} \left( {j,k} \right) \cdot D_{2} \left( {j,k} \right)} ,$$

with values between 0 (minimum) and 1 (maximum). We used *B* to obtain quantitative similarity measures for comparing subtype models, and for comparing bootstrap models to assess within-subtype robustness as in [30].

### Patient stagings

For each subtype model, a patient was assigned to the stage *k* that most closely matched their set of measurements, as in [11, 31]. The possible stage ranged from 0 (i.e., no biomarkers are abnormal) to 12 (i.e., all the biomarkers are abnormal). Precise details on patient staging are in the supplementary materials.

A two-tailed Wilcoxon’s rank-sum test with continuity correction was used to compare (pairwise) the time from first MRI to death of patients assigned to distinct stages within the same model. False-discovery rate was used to correct for multiple comparisons within each subtype model. Significance was determined for adjusted *P* < 0.05.

### Longitudinal validation

Due to the short survival time of CJD and its rapid spread in the brain, we have relatively few follow-ups in our study and several of them are unchanged with respect to baseline. We evaluated the consistency of patient staging with the available longitudinal measurements of sCJD patients: we identified 151 patients with a first follow-up MRI scan, 44 of them having also a second follow-up. For each subtype, we compared first MRI (baseline) stage with first and second follow-up stage(s) across patients: the staging system is longitudinally consistent if patients’ stages at follow-up increase or remain stable with respect to the stages identified by the model at first MRI [11, 31].

### Correlation of in vivo model with postmortem end-stage pathology

In six sCJD subtypes, we quantitatively correlated postmortem pathology burden of eight brain regions retrieved from a study previously reported by our own lab at NPDPSC [21] with the position in the orderings obtained by the event-based model from in vivo MRI data. We used a robust linear regression model to correlate the average neuropathological score from [21] and the average position in the event-based model sequence of the eight regions of interest that are common to both studies. We chose a robust regression model to properly handle the presence of outliers, that usually introduce bias in the estimation when using traditional regression approaches [29].

## Results

### Patients

**Fig. 1:**
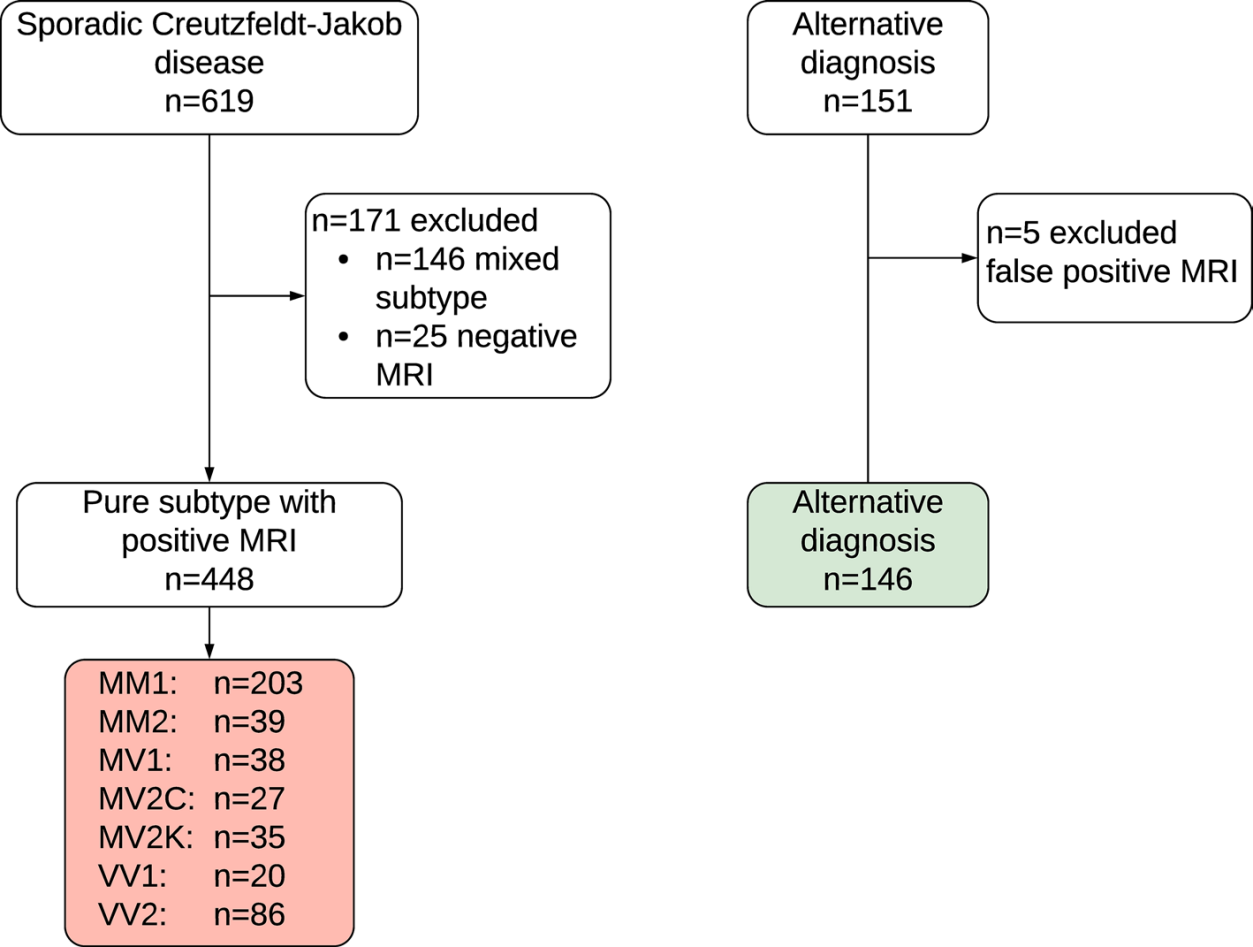
Flowchart of the stepwise characterization of the study subjects. The numerosity of subjects is indicated in each box. Coloured boxes show the groups of subjects included in the event-based model

From the collection of 619 sCJD patients with DWI and autopsy-confirmed diagnosis, 448 subjects with pure subtype and positive MRI were selected and subdivided in 7 groups according to their molecular subtype (pairing of PRNP129 genotype and PrP^D^ type) or, in the case of sCJD-MV2K and -MV2C, according to their histopathological phenotype (histotype) (Fig. 1). Demographic data of the selected sCJD patients are reported in Table 1. We included as controls 146 subjects with autopsy-confirmed diagnosis of non-prion disease. Diffusion MRI from patients belonging to each of the seven “pure” molecular subtypes underwent detailed analyses according to the event-based model to generate diagrams representing anatomical locale of the epicentre and subsequent propagation of the pathological process.

**Table 1:** Demographics of the sCJD patients per subtype

sCJD subtype	Patients No	Patients with multiple MRI, No	Age at MRI, median (IQR) years	Time from symptoms onset to first MRI, median (IQR) days	Time from first to last MRI, median (IQR) days	Time from last MRI to death, median (IQR) days	Time from onset to death, median (IQR) months
MM1	203	75	66 (60–73)	47 (30–67)	14 (6–26)	19 (12–29)	2.6 (2.0–3.4)
MM2	39	12	64 (58–73)	82 (42–235)	66 (46–92)	249 (41–452)	12.4 (5.6–22.6)
MV1	38	15	67 (57–71)	73 (43–114)	22 (10–35)	28 (20–204)	3.9 (2.5–10.3)
MV2C	27	9	65 (62–69)	140 (48–277)	121 (33–207)	147 (80–422)	16.9 (9.1–25.6)
MV2K	35	11	65 (60–69)	158 (86–318)	55 (20–112)	97 (56–199)	11.5 (7.6–15.8)
VV1	20	1	58 (42–69)	123 (48–152)	97 (97–97)	43 (43–43)	7.6 (4.9–12.5)
VV2	86	28	66 (60–62)	99 (61–132)	35 (21–53)	46 (36–57)	5.0 (4.1–6.7)

*sCJD* Creutzfeldt–Jakob disease, *DWI* diffusion weighted imaging, *MRI* magnetic resonance imaging, *IQR* interquartile range

### Comparative analysis of sCJD-MM1 and sCJD-VV2 subtypes

**Fig. 2:**
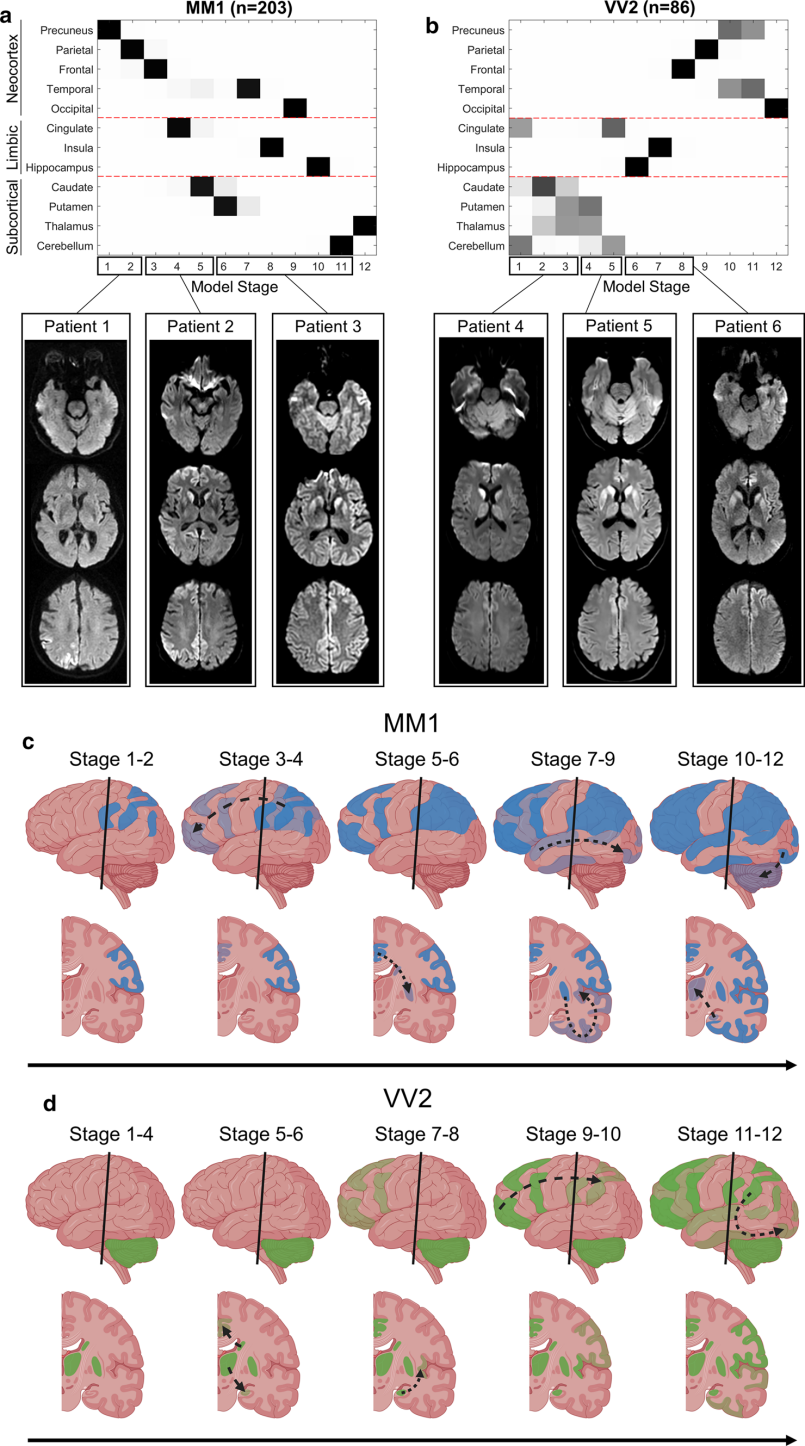
Event-based model of DWI abnormality propagation in sCJD-MM1 and sCJD-VV2 subtypes. **a–b** Diagrammatic representation and diffusion-weighted images (DWI, *b* = 1000) of patients at three different model stages of lesion propagation in sCJD-MM1 (**a**) and -VV2 (**b**). Squares in the diagrams represent the order of appearance of DWI abnormalities (sequence left-to-right; confidence in grey scale) in 12 brain regions (vertical axis) from the neocortex, limbic system, and subcortical regions. In patient 1, DWI hyperintensity are present only in the right precuneus and parietal cortex (stage 2); in patient 2, lesions have propagated to the caudate heads and right anterior frontal cortex (stage 5); in patient 3, abnormalities have propagated to putamina, left temporal and occipital cortices, insula, hippocampus and cerebellum (stage 11); in patient 4, DWI hyperintensities are present in the cerebellum, caudate and thalami (stage 3); in patient 5, lesions have propagated to putamina and cingulate (stage 5); in patient 6, abnormalities have propagated to the left hippocampus, insula, and anterior frontal cortex (stage 8). **c–d** Pictorial representation of prion lesions propagation on the brain surface (upper row) and in coronal slices (lower row) in sCJD-MM1 (**c**) and -VV2 (**d**). Colour intensity represents lesion severity; the dashed curved arrows indicate the direction of the lesion propagation; the vertical black lines indicate the level of the coronal slices

The diagrams in Fig. 2 show that almost opposite sequences of lesion propagation were found in sCJD-MM1 (neocortex to subcortical structures) and -VV2 (subcortical to neocortex), the two most common subtypes, which respectively account for 34% (213/619) and 15% (95/619) of cases in our collection.

In the -MM1 subtype, the precuneus is the earliest region with detectable imaging abnormalities (epicentre) (Fig. 2a). Lesions propagated through the parietal then frontal cortices, followed by the striatum. The cerebellum and thalamus were the last regions to be affected.

In contrast to -MM1, the epicentre in -VV2 is most likely in the cerebellum, with the model suggesting that the cingulate gyrus, the striatum and thalamus, are among the earliest regions affected (Fig. 2b). The neocortex became abnormal after subcortical regions, with neocortical lesions propagating in the opposite direction to -MM1, i.e., frontal to parietal and eventually occipital cortex.

Figure 2 also illustrates the progression of lesion propagation in images. The DWIs of three -MM1 subjects (Fig. 2a) show the progression of signal hyperintensities from the precuneus to the striatum, and eventually to the cerebellum. The DWIs of three -VV2 subjects (Fig. 2b) illustrate that the epicentre is in subcortical regions with propagation to the limbic structures, the frontal cortex and eventually to the cortex of the other lobes. The cartoons illustrate the opposite sequences of lesion propagation in -MM1 (Fig. 2c) and -VV2 (Fig. 2d).

### Less common subtypes with PRNP129 homozygosity: sCJD-MM2 and -VV1

**Fig. 3:**
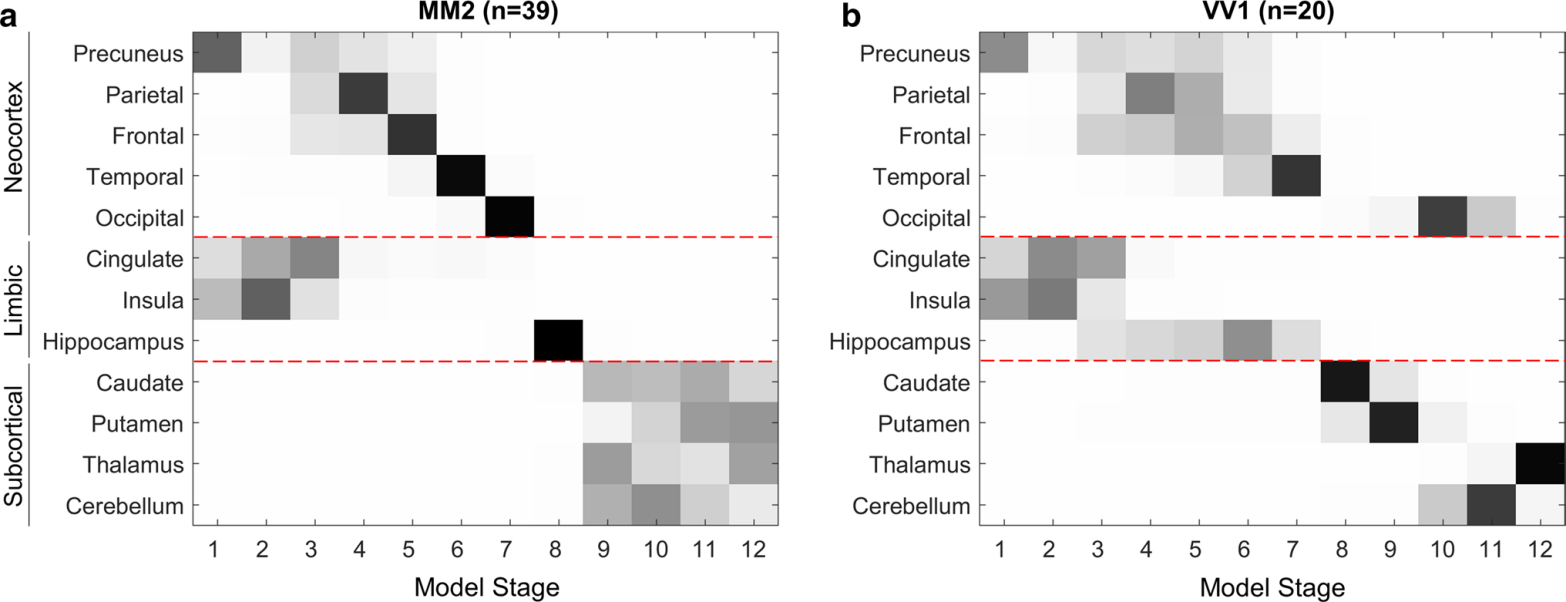
Event-based model of DWI abnormality propagation in sCJD-MM2 and sCJD-VV1 subtypes. **a**, **b** Squares in the diagrams represent the order of appearance of DWI abnormalities (sequence left-to-right; confidence in grey scale) in 12 brain regions (vertical axis) from the neocortex, limbic system, and subcortical regions. The epicentre is in the precuneus. DWI abnormality in the insula and cingulate precede the other neocortical regions and eventually subcortical regions

Figure 3 shows the sequences of lesion propagation in sCJD-MM2 and -VV1. As expected, the sequences of DWI abnormality propagation bear a clearer resemblance to those of -MM1 (*B* = 0.22 for -MM2 and *B* = 0.59 for -VV1) than to those of -VV2 (*B* = 0.03 and *B* = 0.09, respectively). The precuneus is the most likely epicentre in both the -MM2 and -VV1 subtypes, although the model suggests that, unlike -MM1, lesions in the cingulate and insular cortices are among the earliest to appear. Beyond this, the overall -MM1-like pattern of lesion propagation from the neocortex to subcortical regions is maintained in both -MM2 and -VV1 subtypes.

### Less common sCJD subtypes with PRNP129 heterozygosity: sCJD-MV2C, -MV2K and -MV1

**Fig. 4:**
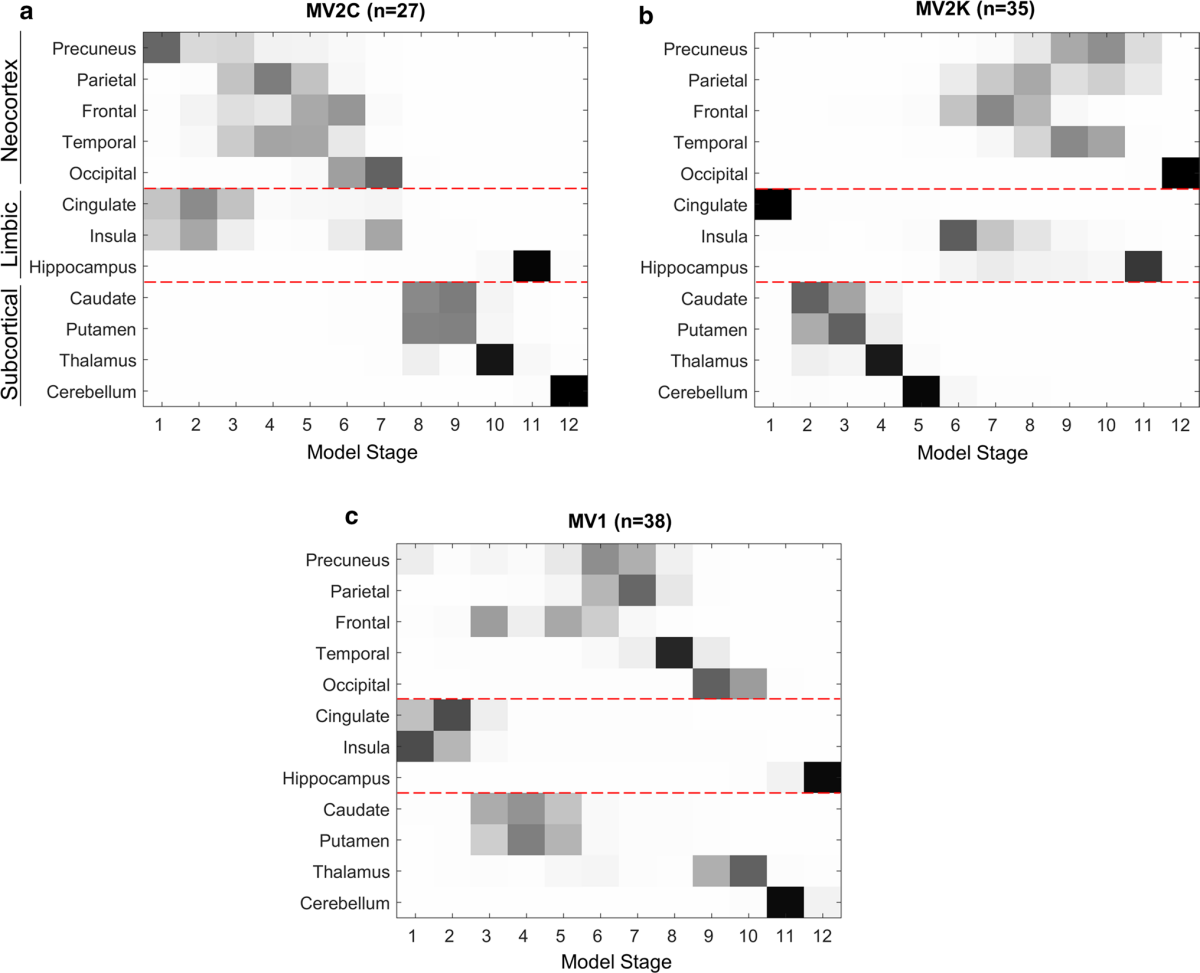
Event-based model of DWI abnormality propagation in heterozygote subtypes. Squares in the diagrams represent the order of appearance of DWI abnormalities (sequence left-to-right; confidence in grey scale) in 12 brain regions (vertical axis) from the neocortex, limbic system, and subcortical regions. **a** In sCJD-MV2C, the epicentre is located in the precuneus as well as in its phenocopy -MM2. Then DWI abnormalities propagate to cingulate, insula that precede the other neocortical regions. **b** On the contrary, in -MV2K, the epicentre is located in the cingulate, then DWI abnormalities propagate to the striatum, thalamus, cerebellum and insula, and eventually to the neocortex. **c** In -MV1 the epicentre is in the limbic structures. Then DWI abnormalities propagate to the striatum that precedes the neocortex and, eventually propagate to thalamus, cerebellum and hippocampus

In general, the order of DWI abnormality propagation was more variable in three heterozygous subtypes. Figure 4a shows that the propagation profile of sCJD-MV2C, which is considered the histopathological phenocopy of -MM2 (Fig. 3a), shares the precuneus epicentre and the general propagation pathway throughout the cerebral cortex, ultimately involving subcortical regions. Quantitatively, the similarity coefficient between -MM2 and -MV2C is high (*B* = 0.60).

Figure 4b shows the propagation profile of -MV2K, which is known to have phenotypical similarity with -VV2 (Fig. 2b). For example, at postmortem examination, -MV2K differs minimally from -VV2—mostly by having strong presence of prion amyloid plaques or kuru plaques in the cerebellum that are invisible to DWI—but the topography of spongiform degeneration is similar [34]. Our results confirm this similarity, showing that the caudal-rostral sequence of lesion propagation that characterises -VV2 is replicated in -MV2K with a high similarity index (*B* = 0.65). The primary, albeit minor, difference is that our models suggest that the cingulate is more likely to contain the epicentre in -MV2K than in -VV2, where the cerebellum seems more likely.

Figure 4c shows the lesion propagation profile of -MV1, which is considered to be indistinguishable from -MM1 based on histopathology [21, 34]. Unexpectedly, our model suggests a lesion propagation pattern that more closely resembles features of -VV1 than -MM1 (*B* = 0.50 for -VV1; *B* = 0.31 for -MM1). For example, in both -MV1 and -VV1, our models suggest that the epicentre is located in the limbic structures (insula, cingulate or both), rather than in the precuneus as in -MM1. Additionally, our -MV1 and -VV1 models estimate that lesion propagation to the striatum precedes cerebral cortical involvement (opposite to -MM1), with frontal lesions preceding parietal lesions (opposite to -MM1, but similar to -MV2K and -VV2).

All subtype model positional variance diagrams (Figs. 2, 3, 4) were individually validated by bootstrapping (Fig. S1, online resource), confirming the robustness of the maximum likelihood sequences obtained for each subtype (similarity *B* ≥ 0.87 for all models).

### Patient staging: time from 1st MRI to death and longitudinal consistency

In each subtype, we systematically compared average interval times from 1st MRI to death between patient groups stratified by model stage, a surrogate for the extent of lesion spread. After correcting for multiple comparisons, only one group difference in sCJD-MM1 reached statistical significance (adjusted *P* < 0.05): patients at stages 3 and 4 had significantly longer interval times from 1st MRI to death (74 ± 45 and 95 ± 55 days, respectively) than those at later stages (from 24 ± 10 to 37 ± 18 days) (Table S1, online resource).

**Fig. 5:**
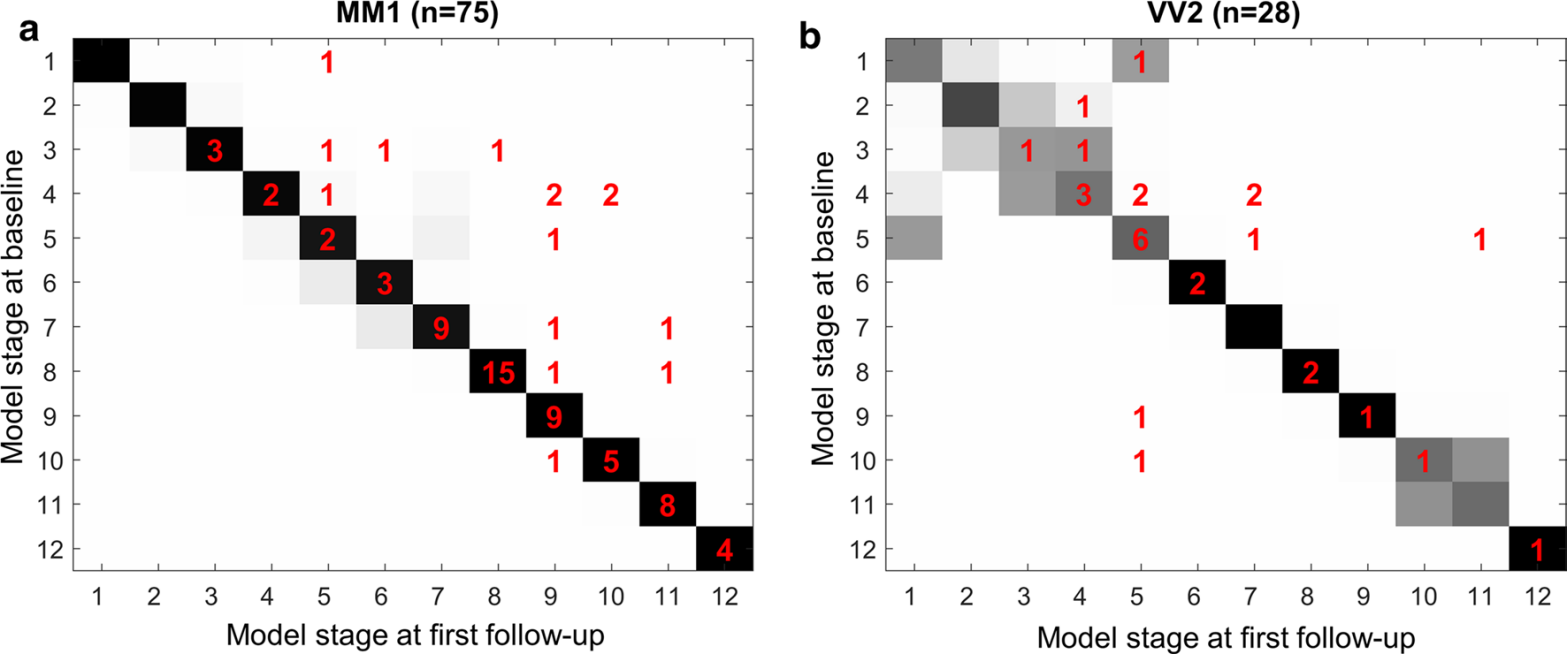
Longitudinal consistency of patient staging in the two most common sCJD subtypes. **a** sCJD-MM1. **b** sCJD-VV2. Diagrams compare stages at baseline (y-axis) and at first follow-up (x-axis) of the same patients according to the event-based model. Each data point (x,y) is represented by an integer (coloured in red) corresponding to the number of individuals contributing to that combination of (follow-up, baseline) stage. For example, in (a), the model assigned one sCJD-MM1 patient to stage 1 at baseline and to stage 5 at follow-up. The staging is longitudinally consistent for the subjects whose coordinates are on or above the diagonal and/or within the grey shaded area, that represents the model confidence in identifying the sequence as shown in the diagrams of Fig. 2a and b: the darker the square, the higher the confidence

Figure 5 shows the longitudinal self-consistency of the -MM1 and -VV2 models, which was excellent. At first follow-up, patient stage either increased or remained unchanged for 99% (74/75) and 93% (26/28) of individuals in -MM1 and -VV2, respectively. Excellent longitudinal self-consistency was also obtained at second follow-up (33/34, 97%; Fig. S2, online resource), as well as for the models of the other subtypes at first (47/48, 98%) and second follow-up (10/10, 100%).

### Correlation of in vivo model with postmortem end-stage pathology

**Fig. 6:**
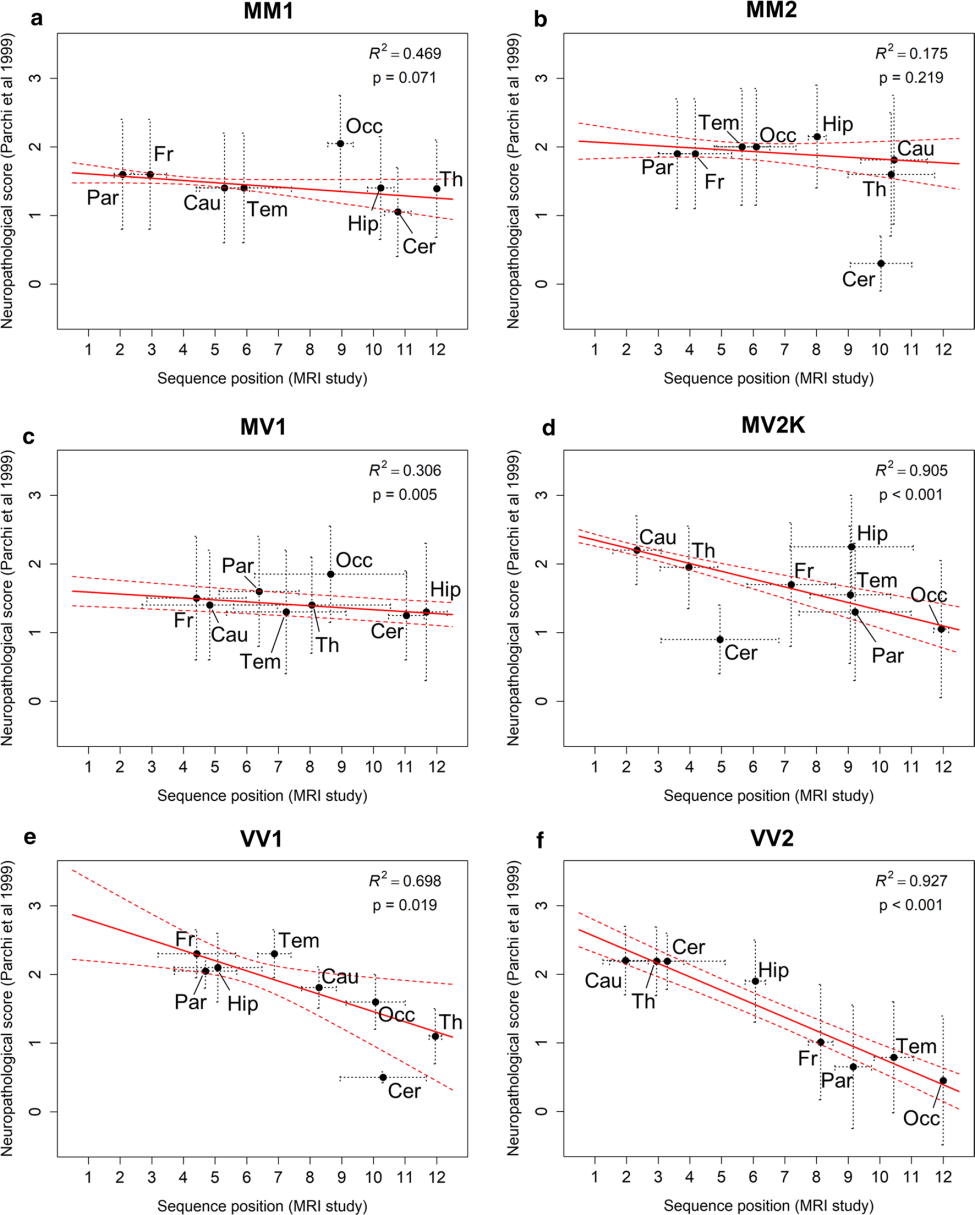
Correlation between our estimated lesion propagation sequences and semi-quantitative neuropathology scores according to sCJD molecular subtype. **a** sCJD-MM1. **b** sCJD-MM2. **c** sCJD-MV1. **d** sCJD-MV2K. **e** sCJD-VV1. **f** sCJD-VV2. Dots represent mean values and error bars represent the standard deviations. Red solid lines represent the fitting of the robust regression model, and red dashed lines are the corresponding 95% confidence intervals. Data points outside the confidence bands are considered outliers. The corresponding region for each data point is indicated with an abbreviated label (*Par* parietal lobe, *Fr* frontal lobe, *Tem* temporal lobe, *Occ* occipital lobe, *Hip* head of hippocampus that includes the entorhinal cortex, *Cau* caudate, *Th* thalamus, *Cer* cerebellum). Positions in the sequence of each event-based model were computed from the MRI data of the present study considering the first MRI study of the patients (203 sCJD-MM1, 39 -MM2, 38 -MV1, 35 -MV2K, 20 -VV1, and 86 -VV2). The neuropathological scores were extracted from the figure of Parchi et al. 1999 [21] (patient numbers: 111 sCJD-MM1, 5 -MM2, 5 -MV1, 19 -MV2K, 3 -VV1, and 30 -VV2) and were originally computed by averaging three scores that measured spongiosis (from 0 to 4), astrogliosis (from 0 to 3), and neuronal loss (from 0 to 3) for each brain region examined

Figure 6 shows a comparison of our in vivo lesion propagation ordering with the end-stage neuropathological lesion profiles described in [21], for the eight brain regions in common to both studies. An inverse correlation was found for all subtypes, with strong and significant correlation for sCJD-VV2 (*R*^2^ = 0.927, *p* < 0.001), -MV2K (*R*^2^ = 0.905, *p* < 0.001), -MV1 (*R*^2^ = 0.306, *p* = 0.005) and -VV1 (*R*^2^ = 0.698, *p* = 0.019). These results support the conclusion that brain regions appearing abnormal on DWI at earlier stages according to the event-based model tend to have higher lesion scores at neuropathological examination in these subtypes. On the contrary, the correlation was not significant for -MM1 (*R*^2^ = 0.469, *p* = 0.071) and -MM2 (*R*^2^ = 0.175, *p* = 0.219).

## Discussion

Current notions of prion propagation in sporadic CJD have been informed by neurological signs observed at diagnosis or on basic comparisons of lesion topography at autopsy in small samples of cases having variable disease duration [3, 26]. Our study shows for the first time by direct in vivo observation that the histopathological process in sCJD is subtype-specific both in terms of epicentre and propagation profile. The epitome of this scenario is represented by the sCJD-MM1 and -VV2 subtypes where our data show that the histopathological process propagates along the rostrocaudal axis in opposite directions. In -MM1, the process is first detected in the precuneus and progressively invades other neocortical regions, striatum and, lastly, cerebellum and thalamus. In -VV2, the epicentre is located in the cerebellum (and possibly the cingulate cortex and caudate) while the neocortex is the last to be affected.

We believe that these advances in knowledge are important both conceptually and clinically since they conclusively show that sCJD consistently begins as a focal condition and propagates to the other brain regions in a subtype-specific manner. This study shows that propagation of spongiform degeneration is predictable, also in a sporadic neurodegenerative condition where the disease process by definition is considered to be spontaneous and idiopathic. At a practical level, the resolution of the model and the new awareness of focal DWI signal hyperintensity is likely to enable earlier MRI diagnosis and in vivo staging of sCJD in individual subtypes.

Considerable information was provided also by our models of the rare subtypes, albeit with a lower degree of confidence likely due to the smaller patient numbers. In the “neocortex first” rare subtypes (sCJD-MM2, -MV2C, and -VV1), the similarity of our models concurs with the similarity of the corresponding postmortem lesion profiles observed in prior pathological work on smaller cohorts [21]. All four “cortical” subtypes share an epicentre in the precuneus, which, according to our data, overall is the most common epicentre in sCJD. Notably, however, the three rare subtypes diverged from the propagation profile of the main “neocortex first” -MM1 subtype by having earlier involvement of the limbic cortex. Thus, the early, exclusive participation of parietal and frontal cortices is unique to -MM1. Future work will leverage recent developments enabling simultaneous delineation of subtypes and progression patterns [30] to automatically uncover data-driven sCJD subtypes with distinct progression patterns, and subsequently relate them to the traditional subtypes. This provides enhanced statistical power, because similar subtypes are automatically grouped into a single data-driven subtype, and may reveal novel heterogeneity within each sCJD subtype.

A surprising and original finding of this study was the clear difference in epicentre location and propagation pattern between sCJD-MV1 and -MM1, which, based on postmortem examination, are considered histopathological phenocopies [34]. As our cross-sectional data show, the trajectories lesion propagation may have converged at later stages and in particular at time of autopsy. Furthermore, at variance with -MM1, -MV1 was characterized by consistent abnormality of cingulate and insular cortices that is often ignored in lesion profiling used to compare the histopathological phenotypes in sCJD subtypes [20, 21]. Combined, these observations underline the limitations of phenotypic determination based only on postmortem examination and introduce the concept that substantial phenotypic heterogeneity might occur in the early stages of the disease becoming blurred terminally. We also found that the -MV1 propagation pattern is intriguingly close to that of -VV1, and that other features are shared with -MV2K and -VV2 subtypes. These additional findings raise the question as to whether the diversity of the early propagation pattern between -MV1 and -MM1 is related to the PRNP129 MV heterozygosity which results in the conversion of both PrP^D^− 129 M and − 129 V in -MV1 [16].

An additional major contribution of our study is that the fine-grained pattern of lesion propagation provides a template for in vivo staging of the disease process and subtype diagnosis. In the most common sCJD–MM1 subtype, we show that the extent of lesion propagation can be predictive of patient survival time. In particular, -MM1 patients with exclusive parietal and frontal involvement have more than double the time from 1st MRI to death of -MM1 patients with lesions extending to other brain regions. Such an association was missing in the other subtypes (after correcting for multiple comparisons), which we hypothesise is due to unknown within-subtype heterogeneity in sCJD lesion propagation: this is the subject of planned future work. The early involvement of subcortical regions and sparing of the cortex, a unique characteristic of -VV2 and -MV2K, might be developed into a diagnostic biomarker for the identification at the earliest stages of the disease in these two subtypes. Another feature of -VV2 and -MV2K, shared also by -MV1 subtype, is the earlier involvement of frontal with respect to parietal cortical ribbon: this feature could be used to discriminate these subtypes from -MM1, -MM(V)2C and -VV1, that have a reversed ordering of involvement of the two cortical regions. Furthermore, the lack of early involvement of the cingulate and insular cortices might distinguish -MM1 from the other cortical variants. The resolution and reliability of the event-based model are likely to provide the neuroradiologists with advancements in knowledge that may enhance early diagnosis of sCJD and of specific subtypes with MRI.

Three longitudinal studies have previously addressed the evolution of DWI abnormalities in CJD [7, 17, 23]. All of them suffer from two key limitations: (i) they rely on predefined a priori stages for assessing lesion propagation, leading to a decrease in the resolution; and (ii) they involve substantially smaller datasets, which preclude the analyses of the individual sCJD subtypes. When we apply the methodology of these previous studies and combine all our sCJD cases regardless of the subtype diagnosis, our results replicate those of Eisenmenger and colleagues showing that neocortical lesions precede striatal lesions in the majority of cases since as shown in our individual subtype-based study the “neocortical” subtype cohort (-MM1, -MM2, -VV1, and -MV2C) accounted for 64% (269/448) of sCJD cases examined (Fig. S3, online resource). However, such an approach obviously misses the subtype-driven heterogeneity of the disease propagation process that we have uncovered.

We validated the propagation profiles on available longitudinal data in the sCJD–MM1 and -VV2 subtypes. Lesion accumulation on MRI was consistent with the lesion propagation profiles in at least 93% of cases. Furthermore, we explored correlation between our in vivo lesion propagation profiles and end-stage neuropathology scores at autopsy in [21], finding that brain regions affected earlier on MRI tend to have higher lesion scores at neuropathological examination for most subtypes. While encouraging and supported by earlier studies [4, 5, 13, 21], our finding was not universal across all subtypes, and it is important to note that comparing the DWI-estimated sequence of lesion propagation with postmortem pathology burden is complex and requires careful interpretation. First, there is ambiguity in the link between DWI lesions and the specific underlying pathology (spongiform degeneration, astrogliosis, and PrP^D^ deposition) [9, 12, 14]. Secondly, the variable temporal lag between MRI and autopsy confounds such an analysis because lesions may propagate further—particularly in regions affected later—and pathology in regions affected early may have saturated [2]. The strong correlation found for sCJD-VV2 and -MV2K is in agreement with [2] who showed that histopathology scores and PrP^D^ buildup in -VV2 are much greater in the cortex of patients with longer disease duration as compared with those with shorter duration. On the contrary, the correlation was not significant for sCJD-MM1 and -MM2 subtypes: the former is known for very quick propagation of prion lesions and shorter survival time, whereas the latter for slower propagation and much longer survival time. This suggests that the in vivo DWI lesion propagation profiles in these MM genotypes converge into indistinguishable pathology load at autopsy.

From a clinical perspective, sCJD-MM1 demonstrates a heterogeneous mix of clinical progression (e.g., classic CJD, Heidenhain variant phenotypes) and is more rapidly progressive, making clinical comparisons with the model more difficult for this subtype. However, the early onset of neurocognitive symptoms and myoclonus is in concordance with the model. A better comparison can be made with -VV2, which initially presents with cerebellar and motor symptoms that later evolve to more cortical (e.g., neurocognitive) symptoms and is in concordance with our model of DWI abnormality propagation.

A focus for future work, which we hope our results and findings motivate and enable to happen more quickly, would be to develop more robust and precise progression models and staging systems for all subtypes. We feel that a large international study would be important to increase the chances of recruiting more patients with less common subtypes.

In conclusion, our study provides the first in vivo MRI data-driven models that identify the brain epicentre and propagation of spongiform degeneration as detected by DWI in pure subtypes of sCJD. Subtype-specific epicentre and propagation profiles mirrored the heterogeneity of the sCJD subtypes. This finding was epitomized by sCJD-MM1 and -VV2 where the disease process initiated in the neocortex and cerebellum, respectively, and propagated along the rostrocaudal axis in opposite directions. Variations were also observed between subtypes with indistinguishable histotypes at autopsy. Overall, our findings shed new light upon the pathogenic mechanisms of sCJD. At a practical level, the identification of distinct epicenters and propagation trajectories for the major sCJD subtypes provides a template for in vivo staging of the disease process. Furthermore, the resolution and reliability of our model is likely to enable the early and, possibly, specific diagnosis of the pure subtypes.
